# Barley AGO4 proteins show overlapping functionality with distinct small RNA-binding properties in heterologous complementation

**DOI:** 10.1007/s00299-024-03177-z

**Published:** 2024-03-13

**Authors:** Fabio Miloro, András Kis, Zoltán Havelda, Ágnes Dalmadi

**Affiliations:** 1 Hungarian University of Agriculture and Life Sciences (MATE), Institute of Genetics and Biotechnology, Gödöllő, Hungary; 2 Agribiotechnology and Precision Breeding for Food Security National Laboratory, Plant Biotechnology Section, Gödöllő, Hungary

**Keywords:** ARGONAUTE 4 (AGO4), *Hordeum vulgare*, *Arabidopsis thaliana*, Small RNAs, RNA-directed DNA methylation, Transposable elements, Heat stress

## Abstract

**Key message:**

**Barley AGO4 proteins complement expressional changes of epigenetically regulated genes in Arabidopsis ago4-3 mutant and show a distinct affinity for the 5′ terminal nucleotide of small RNAs, demonstrating functional conservation and divergence**.

**Abstract:**

The function of Argonaute 4 (AGO4) in *Arabidopsis thaliana* has been extensively characterized; however, its role in monocots, which have large genomes abundantly supplemented with transposable elements (TEs), remains elusive. The study of barley AGO4 proteins can provide insights into the conserved aspects of RNA-directed DNA methylation (RdDM) and could also have further applications in the field of epigenetics or crop improvement. Bioinformatic analysis of RNA sequencing data identified two active *AGO4* genes in barley, *HvAGO4a* and *HvAGO4b*. These genes function similar to *AtAGO4* in an *Arabidopsis* heterologous complementation system, primarily binding to 24-nucleotide long small RNAs (sRNAs) and triggering methylation at specific target loci. Like AtAGO4, HvAGO4B exhibits a preference for binding sRNAs with 5′ adenine residue, while also accepting 5′ guanine, uracil, and cytosine residues. In contrast, HvAGO4A selectively binds only sRNAs with a 5′ adenine residue. The diverse binding capacity of barley AGO4 proteins is reflected in TE-derived sRNAs and in their varying abundance. Both barley AGO4 proteins effectively restore the levels of extrachromosomal DNA and transcript abundancy of the heat-activated *ONSEN* retrotransposon to those observed in wild-type *Arabidopsis* plants. Our study provides insight into the distinct binding specificities and involvement in TE regulation of barley AGO4 proteins in *Arabidopsis* by heterologous complementation.

**Supplementary Information:**

The online version contains supplementary material available at 10.1007/s00299-024-03177-z.

## Introduction

Barley (*Hordeum vulgare* L.) is an important crop worldwide both in terms of cultivated area (49 million hectares) and grain yield produced (145 Megatonnes) (‘FAOSTAT 2023’). Its nutritious grain serves as a valuable resource for both human and animal nutrition (Newton et al. 2011). With a diploid (2n) genome and a haploid complement of only seven large chromosomes, barley is a model organism for diverse research purposes (Rotasperti et al. 2020), and is particularly valuable for genetic studies due to its ability to be crossed and cultivated in various climates and environments (Saisho and Takeda 2011). The study of barley provides insights into crop resilience, as exemplified by its domestication over 10,000 years ago, and due to its adaptability in diverse environments, barley has been proposed as a model for adaptation studies (Saisho and Takeda 2011; Dawson et al. 2015; Rotasperti et al. 2020). Additionally, barley plays an important role in the study of polyploid wheat and other Triticeae species (Lü et al. 2015).

Plants face a dynamic and challenging environment in which they may be exposed to drought, salinity, high temperatures, nutrient deficiencies, heavy metals, and ultraviolet radiation (Chinnusamy et al. 2014; Chang et al. 2020). These stresses can significantly reduce yields and are detrimental to agricultural productivity. While extensive research has focused on the signal transduction mechanisms of abiotic stress responses, there is growing evidence that RNA silencing and epigenetic mechanisms play a critical role in these processes (Sahu et al. 2013; Kim et al. 2015b; El-Sappah et al. 2021; Kryuvrysanaki et al. 2021; Tiwari and Rajam 2022). For example, priming allows plants to store stress signals and improve their responsiveness to recurring stress conditions within the same plant, and to exhibit transgenerational effects through epigenetic mechanisms such as histone modifications (Popova et al. 2013; Crisp et al. 2016; Ramakrishnan et al. 2022; Harris et al. 2023).

RNA silencing is a nucleotide sequence-specific gene regulatory mechanism that controls developmental processes, heterochromatin maintenance, as well as responses to abiotic and biotic stress. In plants, small RNAs (sRNAs) are generated from RNA precursors, either partially or perfectly double-stranded RNA (dsRNA), by the action of an RNase III-like nuclease known as DICER-LIKE (DCL). These sRNAs incorporate into another nuclease known as ARGONAUTE (AGO), which uses Watson–Crick base pairing to guide the AGO complex to its target nucleic acids (Baulcombe 2004; Molnar et al. 2011). This nucleoprotein complex down-regulates gene expression either via transcriptional silencing, involving DNA and histone methylation, or through post-transcriptional action, which includes mRNA cleavage or translational inhibition of the target sequence (Ghildiyal and Zamore 2009; Baulcombe 2015; Guo et al. 2016; Hung and Slotkin 2021). This diversity of functions has been made possible by the expansion of gene families encoding RNA silencing components. The individual AGO family members have specific connections to different types of sRNAs, enabling them to operate diverse regulatory pathways (Hutvagner and Simard 2008).

The *Arabidopsis* genome encodes ten AGO proteins which, for the most part, have distinct roles; however, in some cases, their functions overlap. The nature of their functionality is typically reflected by the associated sRNA content (Zilberman et al. 2003; Baumberger and Baulcombe 2005; Qi et al. 2005, 2006; Havecker et al. 2010). AGO proteins consist of the following domains: N-terminal; PIWI/Argonaute/Zwille (PAZ); MID; P-element-Induced WImpy testis (PIWI) domains and two linkers (Hutvagner and Simard 2008). The PIWI domain forms a pocket with the MID domain that recognizes the 5′-end of the sRNA (Zhang et al. 2014a; Liu et al. 2022). Based on their length, and the nucleotide at the 5′-end, sRNAs can be sorted into different AGO proteins. As an example, in the model plant species *Arabidopsis thaliana* AGO1, the key regulator of microRNA (miRNA) pathway, preferentially loads 21-nt sRNAs starting with a uridine (U); whereas, AGO4 and AGO6, which participate in RNA-directed DNA methylation (RdDM), exhibit a preference for 24-nt sRNAs starting with an adenine (A) (Mi et al. 2008).

In plants, RdDM is a process that modulates the chromatin state through 24-nucleotide long small interfering RNAs (24-nt siRNAs), while it is also responsible for directing de novo DNA methylation and transcriptional gene silencing (Wassenegger et al. 1994; Matzke et al. 2002; Gao et al. 2010; Zhang et al. 2013; Matzke and Mosher 2014; Gallego-Bartolomé, 2020). The combined action of plant-specific DNA-DEPENDENT RNA POLYMERASE IV (Pol IV) and RNA-DEPENDENT RNA POLYMERASE 2 (RDR2) generates dsRNA precursors, which are then processed by DICER-LIKE 3 (DCL3) into 24-nt siRNAs (Li et al. 2015; Huang et al. 2021). One strand of the duplex is subsequently loaded onto the AGO4 effector protein and assembled with nascent transcripts produced by RNA polymerase V (Pol V). This process leads to the recruitment of chromatin remodeling factors, including the de novo DNA methyltransferase DRM2 (Domains Rearranged Methyltransferase 2) and histone modifying enzymes. (Wierzbicki et al. 2009; Sigman et al. 2021; Zheng et al. 2021).

AGO4, as the effector protein of RdDM, binds siRNAs associated with repeats and heterochromatic regions, and its mutant phenotype results in the loss of epigenetic modifications at different chromosomal loci (Zilberman et al. 2003; Qi et al. 2006). AGO4 and its paralogs (AGO6 and AGO9) have been extensively studied in *Arabidopsis* using mutants (Havecker et al. 2010; Duan et al. 2015; Wang et al. 2023). Contrarily, in monocotyledons, only rice and maize orthologs have been experimentally studied (Nonomura et al. 2007; Kapoor et al. 2008; Qian et al. 2011; Yang et al. 2013; Li et al. 2019; Zhai et al. 2019; Aubert et al. 2022). Rice was found to have four different members of the AGO4 clade, namely AGO4A, AGO4B, AGO15, and AGO16. Specifically, it was shown that both AGO4A and AGO4B load 24-nt long miRNAs (lmiRNAs) and siRNAs but exhibit a distinct specificity for their loaded sRNAs. While rice AGO4A primarily loads sRNAs with an adenine residue at their 5′ end, AGO4B exhibits no preference for a specific nucleotide at the 5′ end. (Wu et al. 2009, 2010). AGO4 has also been shown to play a role in antiviral and pathogen protection, as an alternative effector of specific miRNAs, and in maintaining genome stability by silencing transposable elements (TEs) (Agorio and Vera 2007; Wu et al. 2010; Scholthof et al. 2011; Brosseau et al. 2016; Panda et al. 2016; Pradhan et al. 2020; Guo et al. 2021). Small TEs and TE fragments located near genes are targeted by RdDM and are typically located in open and accessible euchromatic regions of the genome that facilitate gene expression (Sigman and Slotkin 2016). TEs are a major factor in shaping the epigenetic changes that regulate plant development and stress adaptation (Ito et al. 2011; Ramakrishnan et al. 2021). A substantial fraction of epigenetic alterations in plants are due to the interactions of TEs with the genome. With the probability of their activation under stress conditions, TEs can trigger mutagenic effects and significant genetic variability (Kazazian 2004; Casacuberta and González 2013; Ito et al. 2013). In addition to its role in maintaining the silencing of TEs, RdDM can induce transcriptional silencing of foreign DNA, including novel TE insertions, viral-derived sequences, and transgenes (Chan et al. 2004; Marí-Ordóñez et al. 2013).

In this study, we have identified two active paralogous barley genes and one presumed pseudogene within the AGO4 clade by in silico analysis. We demonstrated different properties in the binding and functionality of sRNAs when introduced into an *Arabidopsis ago4* mutant via heterologous complementation. This approach facilitated the verification of the functionality of the two active barley AGO4 candidate genes in terms of the expressional regulation of RdDM targets, and enabled the discovery that their specific preferences for sRNAs originated from different TE regions.

## Materials and methods

### Plant material and growth conditions

*Arabidopsis thaliana* plants were grown under controlled conditions of 16-h light/8-h dark at 21 °C. *A. thaliana* (Col-0) and *ago4-3* mutant (WiscDSLox338A06) plants were used in this study. The homozygous *ago4-3* mutant was derived from the original WiscDSLox338A06.0 T0 line (Havecker et al. 2010), which was regularly checked by growing seeds on MS agar plates containing phosphinothricin (10 mg/L), assessing the protein level of AtAGO4 using an anti-AtAGO4 antibody (Agrisera, AS09 617), and via PCR amplification of the region of T-DNA inserted approximately 170 bp before translational initiation (Fig. S1A–C). Mixed-stage inflorescences were collected from approximately 7-week-old plants for DNA and RNA extraction.

For heat stress treatment, 1-week-old *Arabidopsis* seedlings grown on agar plates (1/2 MS, 0.8% agar and 0.5% sucrose) were transferred to a growth chamber set to 37 °C for 24 h, and lighting conditions were identical to those used for control plants (21 °C), following a modified protocol described previously (Szádeczky-Kardoss et al. 2022). Bulked seedlings were harvested for DNA and RNA extraction.

*Hordeum vulgare* cultivar Golden Promise plants were grown under a day temperature of 20 °C and a night temperature of 16 °C, with a photoperiod of 16-h light (400 µmol/m^2^/s) and 8-h dark. The main tillers of approximately 3-month-old plants were used to dissect the developing inflorescences, and only those between 15 and 25 mm in length (white anther stage) were collected for RNA extraction.

### Phylogenetic analysis

The full peptide sequences of AGO4-6 clade from *A. thaliana*, *Oryza sativa* ssp. *Japonica,* and *H. vulgare* (*cv.* Morex and *cv.* Golden Promise) were retrieved from Ensembl Plants and UniProt and were aligned using ClustalW. The aligned sequences were used to construct a phylogenetic tree with the neighbor-joining method (Saitou and Nei 1987) and the percentage of replicate trees in which the associated taxa clustered together in the bootstrap test (1000 replicates) is shown above the branches. The tree is drawn to scale, with branch lengths measured in the number of substitutions per site. Evolutionary distances were calculated using the Poisson correction method. Evolutionary analyses were conducted in MEGA11 (Tamura et al. 2021).

### Plasmid construction and plant transformation

All plant expression plasmids were constructed using the pGreen 0029 vector (kanamycin resistance) (www.pgreen.ac.uk). The *AtAGO4* promoter (~ 2500 bp) and terminator (~ 500 bp) sequences, as well as *HvAGO4a* (2766 bp) and *HvAGO4b* (2757 bp) cDNA sequences were amplified using Phusion Hot Start II (Thermo Fisher Scientific), following the manufacturer’s instructions (primers sequences listed on Supplementary Table S1). During cloning, the ATG start codon was removed from both the sequences and the MluI restriction site and the HA epitope tag with the start codon was added at the 5′ end. In addition, both sequences contain a TGA stop codon to which we added an additional TAA stop codon and XbaI restriction site at the 3′ end. The pGreen0029 was first ligated with the *AtAGO4* promoter, followed by ligation to the terminator and finally with the modified cloned cDNA, using the two distinct restriction sites, ensuring their introduction in the correct orientation. The plasmids were first transformed into *Escherichia coli* DH5α competent cells using the heat shock transformation method. *E. coli* were grown in LB plates and liquid media containing 50 mg/L kanamycin, following this plasmids were extracted and their sequence was confirmed via sequencing. All plasmids were transformed into *Agrobacterium tumefaciens* AGL1 strain with electroporation (360 Ω, 25 µF and 2.5 kV) in the presence of pSoup helper plasmid. *A. tumefaciens* was grown on YEB plates and liquid media containing 25 mg/L rifampicin and 50 mg/L kanamycin. A single colony was selected for the transformation of *Arabidopsis ago4-3* mutant (phosphinothricin-resistant) plants using the floral dip method (Zhang et al. 2006). The developing *Arabidopsis* inflorescences were dipped into the *A. tumefaciens* suspension containing 5% sucrose and 0.05% (vol/vol) Silwet L-77 for 1 min. Transformed plants were selected on agar plates (1/2 MS, 0.8% agar and 0.5% sucrose) using phosphinothricin (10 mg/L) and kanamycin (50 mg/L).

### RNA isolation and RT-qPCR

Total RNA was extracted from *Arabidopsis* T3 mixed-stage flowers, seedlings and barley developing inflorescences, using the standard phenol–chloroform method as described previously (Dalmadi et al. 2021).

For RT-qPCR assays, 4 μg total RNA was treated with DNaseI, re-isolated by the phenol–chloroform method, and resuspended in sterile water. 2 µg of DNaseI-treated total RNA and a combination of random hexamer primers and oligo(dT)18 primers were used to synthesize first-strand cDNA using the Maxima H Minus First Strand cDNA Synthesis Kit with dsDNase (Thermo Fisher Scientific) according to the manufacturer’s instructions. qPCR was performed using the Luminaris Color HiGreen qPCR Master Mix (Thermo Fisher Scientific) and performed on a LightCycler 96 Instrument (Roche) real-time PCR machine. Data were obtained from three independent biological replicates and were normalized to *AtUBC9*, *AtACT2,* and *AtPP2AA3* using LigthCycler 96 software (2^−∆∆Ct^ method). Graphs and statistical analysis were generated using GraphPad Prism 8. For primer sequences, please see Supplementary Table S1.

### Protein extraction and western blotting

Mixed-stage flowers were homogenized in extraction buffer (0.1 M glycine–NaOH, pH 9.0, 100 mM NaCl, 10 mM EDTA, 2% SDS), an equal volume of 2 × Laemmli buffer was added. Following this, samples were boiled for 5 min, and centrifuged at 20,000 × g at 4 °C for 5 min to remove debris.

*Arabidopsis* protein samples were run on 8% SDS-polyacrylamide gel, then blotted onto an Amersham Hybond P 0.45 PVDF blotting membrane (GE Healthcare) using wet tank transfer, followed by western blot analysis. Antibodies were used in following concentrations: anti-HA-peroxidase 1:2000 (rat 3F10, Roche), anti-actin (plant) 1:2000 (mouse 10-B3, Sigma-Aldrich), anti-AtAGO4 1:5000 (rabbit AS09 617, Agrisera), and anti-BiP 1:10,000 (rabbit AS09 481, Agrisera). The secondary antibodies used were goat anti-rabbit HRP-conjugated (for anti-AtAGO4 and anti-BiP, AS09 602, Agrisera) and goat anti-mouse HRP-conjugated (for anti-actin, A4416, Sigma-Aldrich). Blocking was carried out in 5% milk powder in PBST for 1 h. Primary antibodies were incubated in 1% non-fat milk powder in PBST for 1 h, and secondary antibodies were diluted with PBST and incubated on the membrane for 1 h. Blots were washed three times for 5 min with PBST between the two solutions and finally developed using High Clarity Western ECL (Biorad) on ChemiDoc™ MP Imaging System (Biorad). Volume intensity of the signal was quantified using Image Lab 6.1; protein signals were normalized to either actin or BiP.

### Chop-qPCR analysis

For the Chop-PCR assay, genomic DNA was extracted from *Arabidopsis* T3 mixed-stage inflorescence using the ZenoGene Plant DNA Purification Kit (ZENON Bio), according to the manufacturer’s instructions. Quantification of genomic DNA was performed using a Nanodrop ND-1000 spectrophotometer. To perform the methylation-sensitive enzymatic digestion of DNA, MspJI (New England BioLabs), a modification-dependent endonuclease that specifically recognizes cytosine modifications including C5-methylation (5-mC) and C5-hydroxymethylation (5-hmC), was used to digest genomic DNA (Zheng et al. 2010; Zhang et al. 2014b; Dasgupta and Chaudhuri 2019). This enzyme can cleave asymmetric methylation sites (CHH), in addition to CpG and CHG regions.

The reaction was performed in a 30 µL reaction mix containing 10× rCutSmart™ Buffer (New England BioLabs), 1 µL of MspJI enzyme, 1 µL of Enzyme Activator Solution (New England BioLabs) and 600 ng of genomic DNA over a period of for 4 h at 37 °C. MspJI was omitted exclusively in the control reaction. After heat inactivation, 1 µL of digested and undigested genomic DNA was used as a template for the Chop-qPCR assay. Measurements were prepared using the Luminaris Color HiGreen qPCR Master Mix (Thermo Fisher Scientific) and performed using a LightCycler 96 Instrument (Roche) real-time PCR machine. Data were obtained from three independent biological replicates and were normalized to undigested *AtSN1* using LigthCycler 96 software. Graphs and statistical analysis were generated using GraphPad Prism 8. For primer sequences, please see Supplementary Table S1.

### Relative copy number assessment of transposable elements

Genomic DNA was extracted from *Arabidopsis* non-treated (NT) and heat-stressed (HS) seedlings using the ZenoGene Plant DNA Purification Kit (ZENON Bio) according to the manufacturer’s instructions. Quantification of genomic DNA was performed using a Nanodrop ND-1000 spectrophotometer.

The relative quantification of *Ty1*/*copia*-like retrotransposon *ATCOPIA78* (*ONSEN*) copies was performed using non-treated *Arabidopsis* wild-type DNA as a control, and in relation to its quantification consisting of eight *ONSEN* copies in the Col-0 ecotype genome (Ito et al. 2011, 2013; Hayashi et al. 2020; Nozawa et al. 2022), all relative amounts of the other non-treated and heat-stressed samples were measured using qPCR. Analysis by qPCR was performed using the Luminaris Color HiGreen qPCR Master Mix (Thermo Fisher Scientific) with 20 ng of genomic DNA per reaction and the LightCycler 96 Instrument real-time PCR machine (Roche). Data were obtained from three independent biological replicates and were normalized to *AtUBC9* using LightCycler 96 software. For primer sequences and the list of the eight *ONSEN* copies locus names, please see Supplementary Table S1.

### RNA-seq and analysis

RNA extracted from developing barley inflorescences was quantified using the Qubit RNA HS Assay and a quality check was carried out using the LabChip GX Touch Nucleic Acid Analyzer with the DNA 5K/RNA/CZE chip, resulting in an RNA quality score of 10 in all analyzed samples.

The NEXTFLEX^®^ Rapid Directional RNA-Seq Kit 2.0 was used to prepare libraries for Illumina sequencing instruments according to the manufacturer’s protocol. Briefly, poly-A-containing mRNA was purified from 1250 ng total RNA using the Nextflex polyA beads 2.0 kit. The purified mRNA was fragmented and the first and second cDNA strands were synthesized. Adapter ligation (NextFlex Unique Dual Index Barcodes) and amplification by PCR were then performed. The XMark HT chip (Labchip GX Touch Nucleic Acid Analyzer) was used for the quantification and the quality control of the purified cDNA libraries, which were then normalized and pooled equimolarly. The flow cell was loaded onto the Illumina NovaSeq 6000 for paired-end sequencing using the S4 Reagent Kit v1.5 (300 cycles) according to the manufacturer’s instructions.

Analysis was performed on paired-end fastq raw data with salmon (version 1.10.1) using the Morex V3 (GCA_904849725) reference genome, transcripts fasta file and a file containing a mapping of transcripts to genes (GTF). This analysis enabled the obtainment of the number of reads mapped on each transcript as well as the relative abundance of the transcript in Transcripts per Million (TPM).

### AGO4-associated sRNA library preparation and analysis

For AGO4-associated sRNAs, mixed-stage inflorescences of 7-week-old *Arabidopsis* plants from 3 independent T3 complementation lines of *HvAGO4a* and *HvAGO4b* were collected and processed using the Dynabeads Protein G Immunoprecipitation Kit (Thermo Fisher Scientific) with the anti-HA-peroxidase antibody (rat 3F10, Roche) according to the manufacturer’s instructions. The immunoprecipitated fractions were eluted in 40 μL, from which 30 μL was used for RNA purification and 10 μL for protein samples. RNA extraction was performed using the standard phenol–chloroform method and used to generate cDNA libraries for sRNA-IP sequencing using the Truseq Small RNA Library Preparation Kit (Illumina) using a modified protocol described previously (Czotter et al. 2018). The libraries were sequenced on an Illumina NextSeq 500 system using the NextSeq 500/550 v2.5 sequencing reagent kit. The sequencing mode was single-end 50 bp reads. Protein samples were used to check for the corresponding transgenic AGO4 by western blot using anti-HA-peroxidase antibody (rat 3F10, Roche). Contamination of total proteins was checked by visualization provided by the TGX technology of BioRad gels (Fig. S2).

Raw data of AtAGO4 sRNA-IP sequencing were retrieved from NCBI (SRX11482423, SRX11482424, SRX11482425) (Sigman et al. 2021). Analysis of the sRNA-IP sequencing was performed using the Galaxy platform (Afgan et al. 2022) for quality control, trimming, mapping to the *A. thaliana* reference genome (TAIR10.1) using hisat2 (version 2.2.1) (Kim et al. 2015a) and then mapping to categories of genomic sequences using the following sRNAPipe (Pogorelcnik et al. 2018) pipeline (version 1.1.1): TEs, gene transcripts, microRNAs (miRNAs), small nuclear RNAs (snRNAs), ribosomal RNAs (rRNAs), and transfer RNAs (tRNAs). sRNAPipe allowed for the selection of the size range (18–27 nt) of the sRNAs and the generation of “bonafide” reads that match the genome-mappers, excluding reads that map miRNAs, rRNAs, tRNAs or snRNAs for the normalization as RPM (Reads Per Million) and RPKM (Reads Per Kilobase per Million mapped reads).

Visualization of sRNAs mapped to chromosomes or loci was performed using IGV (https://software.broadinstitute.org/software/igv/) after alignment. Protein alignments were found using ESPript 3 (Robert and Gouet 2014). GraphPad Prism 8 was used to generate graphs and for statistical analysis.

## Results

### Bioinformatic analysis and the determination of expression levels of the putative *AGO4* genes in barley

Analysis of the whole barley genome (both Morex V3 and Golden Promise v1) identified four putative candidate genes belonging to the AGO4-AGO6 clade using sequences from *A. thaliana* and rice. Following a BLAST search on Ensembl Plants, the translated protein sequences of these four genes were analyzed using InterPro (Paysan-Lafosse et al. 2023) to determine which protein family they belonged to and whether they contained the characteristic domains of the functional AGO proteins. To keep the nomenclature consistent, the corresponding proteins were named based on the phylogenetic relationship to their *Arabidopsis* and rice counterparts.

**Fig. 1:**
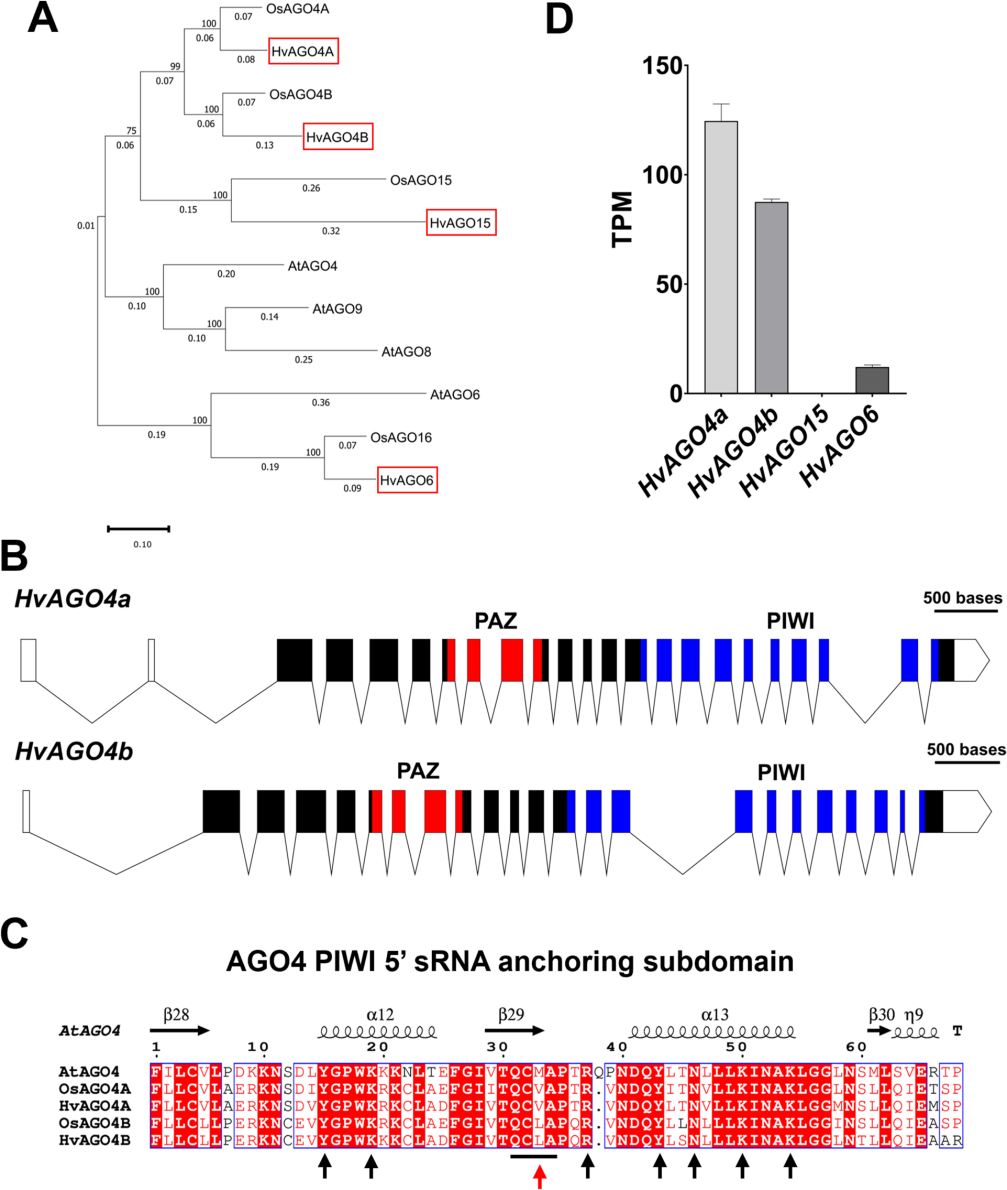
**A** Phylogenetic tree of AGO4-clade protein sequences from *Arabidopsis thaliana*, *Oryza sativa *subsp.* japonica* and *Hordeum vulgare* was inferred using Maximum Likelihood method (1000 bootstrap repetitions) and JTT matrix-based model. Barley AGO4-like translated protein sequences were marked with a red square. **B** Visualization of the *HvAGO4a* and *HvAGO4b* gene structure, including the 5′ and 3′ UTR. Solid rectangles and lines indicate exons of the coding regions and introns, respectively. The UTR regions are marked with empty shapes. Within the coding region PAZ and PIWI domains were labeled with red and blue, respectively. The scale bar represents 500 bp. **C** Protein alignment of the PIWI domain region involved in the 5′ sRNA anchoring of the *Arabidopsis*, rice, and barley AGO4 proteins. Alignment was performed with ClustalW and was visualized using ESPript. The vertical black arrows and the black line indicate the amino acids directly involved in the binding domain, while the red arrow indicates the AA showing variations between *Arabidopsis* (dicot) and the monocots. **D** Transcript abundancy of putative AGO4-clade genes in *H. vulgare* cv. Golden Promise (developing inflorescences) expressed in TPM (calculated using Salmon). Error bars represent the mean ± SD, *n* = 3 (color figure online)

According to the phylogenetic tree generated from the AGO4 homologous protein sequences of *A. thaliana*, *O. sativa* ssp. *japonica*, and *H. vulgare*, two main subclades were distinguished, one of which contained the AGO6-like proteins (including HvAGO6), the other included those proteins which show higher similarity to AGO4 (Fig. 1A). Within the latter AGO4 group, additional subclasses were observed corresponding to dicots (AtAGO4, AtAGO8, and AtAGO9) and to monocots (AGO4s and AGO15s). The identified barley protein sequences of the AGO4 group clustered together with OsAGO4A, OsAGO4B, and a candidate pseudogene OsAGO15 (Wu et al. 2010; Trujillo et al. 2018), respectively. The putative orthologous AGO4 proteins from barley showed a higher identity to the corresponding rice proteins (85% and 82% for AGO4A and AGO4B, respectively) than with each other (barley 76% and rice 79%). Sequence analysis revealed that HvAGO4A (HORVU.MOREX.r3.3HG0256890) and HvAGO4B (HORVU.MOREX.r3.1HG0095310) contained 921 and 918 amino acids, respectively, and showed an identical gene structure in terms of coding exon number, and the position of the PAZ and PIWI domains (Fig. 1B). In particular, the analysis of the PIWI domains of the three AGO4 proteins from barley, *Arabidopsis* and rice allowed us to acquire an improved understanding of their similarities and differences (Fig. 1C). Among the subdomains characteristic of the PIWI domain, there is one region responsible for anchoring the 5′-end of the sRNA. The alignment of the AGO4 proteins within this subdomain revealed a high degree of conservation, with nearly all amino acids responsible for anchoring the 5′-end of sRNAs being strictly conserved across the three plants. However, a notable exception was observed at the site formed by four amino acids (QCxA), where the monocot protein sequences exhibited a single amino acid variation that differed from the *Arabidopsis* counterpart, which was characteristic of the inherent differences between the two AGO4 proteins. Interestingly, the identified barley protein sequences share this feature with their rice counterparts, suggesting a similar function for HvAGO4A and HvAGO4B. The same analysis was carried out for AGO6, showing that the location of the anchor site for the 5′-end of the sRNAs was the same as in AGO4 (α12-β29-α13). Moreover, this analysis also demonstrated a high conservation among the sites of the different plants, with the exception of one amino acid in the site formed by four amino acids (QCIx), which was a variation in a different location from that observed in AGO4 (Fig. S3A).

To evaluate the expression level of the three barley genes closely related to the AGO4 clade, RNA-seq was performed on developing barley inflorescences. Within the clade, *HvAGO4a* exhibited the highest expression, followed by *HvAGO4b*, while *HvAGO15* (HORVU.MOREX.r3.7HG0736980) was expressed at an exceptionally low level. Notably, *HvAGO6* (HORVU.MOREX.r3.5HG0468930) expression was significantly lower when compared to the two *AGO4* genes (Fig. 1D).

Unlike rice, the barley *AGO15* gene seems not to be a result of *AGO4a* tandem duplication since it is located on a different chromosome and does not contain the intronic TEs. Nevertheless, like in rice, the expression level of *HvAGO15* was undetectable suggesting that is either a pseudogene or it is only expressed under certain circumstances and/or in special tissue type. The analysis of this gene showed the presence of 6 tandem repeats in exon 1, in frame with the coding region and with putative different start codons (Fig. S3B). We also encountered limitations in PCR amplification of HvAGO15 as a complete gene from both leaf and inflorescence, obtaining only fragments. Due to these characteristics, *HvAGO15* was omitted from the analysis to focus on the two paralogous genes *HvAGO4a* and *HvAGO4b*. These genes were investigated in heterologous complementation in *Arabidopsis* to validate their functionality as members of the AGO4 family.

### Introduction of barley *AGO4* genes into *Arabidopsis* for complementation studies

To demonstrate the functionality of the two putative barley *AGO4* genes, a heterologous complementation assay was designed using the *AtAGO4* promoter and terminator to insert the 5′ HA-tagged version of each of the barley genes in question into *A. thaliana ago4-3* mutants (WiscDSLox338A06) (Fig. S3C). Initially, T0 plants were selected for kanamycin resistance, and from the T1 generation onward, an analysis of the expression of both mRNA and protein levels in the inflorescence was carried out. As no differences were observed at the phenotypic level between wild-type Col-0 and the *ago4-3* mutant (Havecker et al. 2010), transgenic lines of wild-type appearance were selected for further analysis to avoid positional effects of the transgene insertion.

**Fig. 2:**
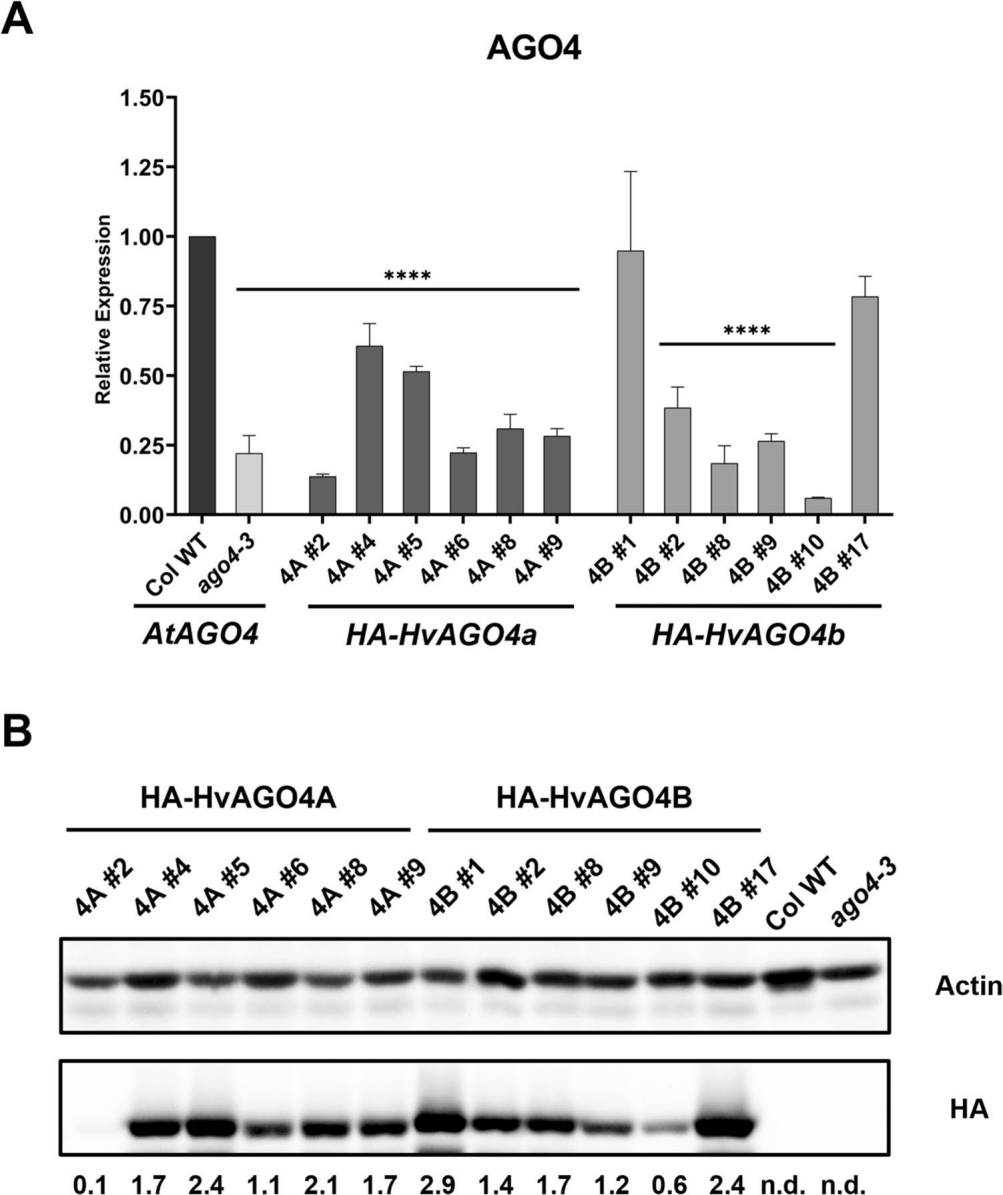
**A** *AtAGO4* and HA-tagged barley *AGO4* gene expression levels determined by RT-qPCR of T1 mixed-stage inflorescences. Data were normalized using *AtUBC9* and *AtACT2*. For individual primer pairs used to detect the three different AGO4 genes see Table S1. The average of 3 independent biological replicates was calculated and statistically significant differences from Columbia wild type (Col WT) are indicated with asterisks (Anova one-way with Dunnett’s post-hoc test, * < 0.05, ** < 0.01, *** < 0.001 and **** < 0.0001). Error bars represent the mean ± SD, *n* = 3. **B** HA-HvAGO4 protein level in mixed-stage inflorescences of T1 transgenic plants. To quantify the HA-HvAGO4, volume intensity of each sample was referred to the corresponding actin signal and was presented as the ratio of HA and Actin signal

Varying levels of transgene expression were observed on an RNA level amongst the transformants using specific primers for the corresponding barley genes. A comparison of the expression of *HA-HvAGO4a* and *HA-HvAGO4b* with the expression of *AtAGO4* in wild-type Col-0 showed that some transformed lines resembled the level of endogenous AGO4, particularly two HvAGO4B lines (#1 and #17) (Fig. 2A). To further compare the AGO4 protein content of the transformant lines, western blot was performed using anti-HA antibody, and a correlation between mRNA and protein levels was observed (Fig. 2B). Subsequently, three lines with prominent AGO4 content were selected for both barley genes, and it was presumed that their different expressional state would enable a transgene dosage dependent analysis of the complementation effect.

### Assessment of the functionality of barley AGO4 in the *Arabidopsis* complementation system

**Fig. 3:**
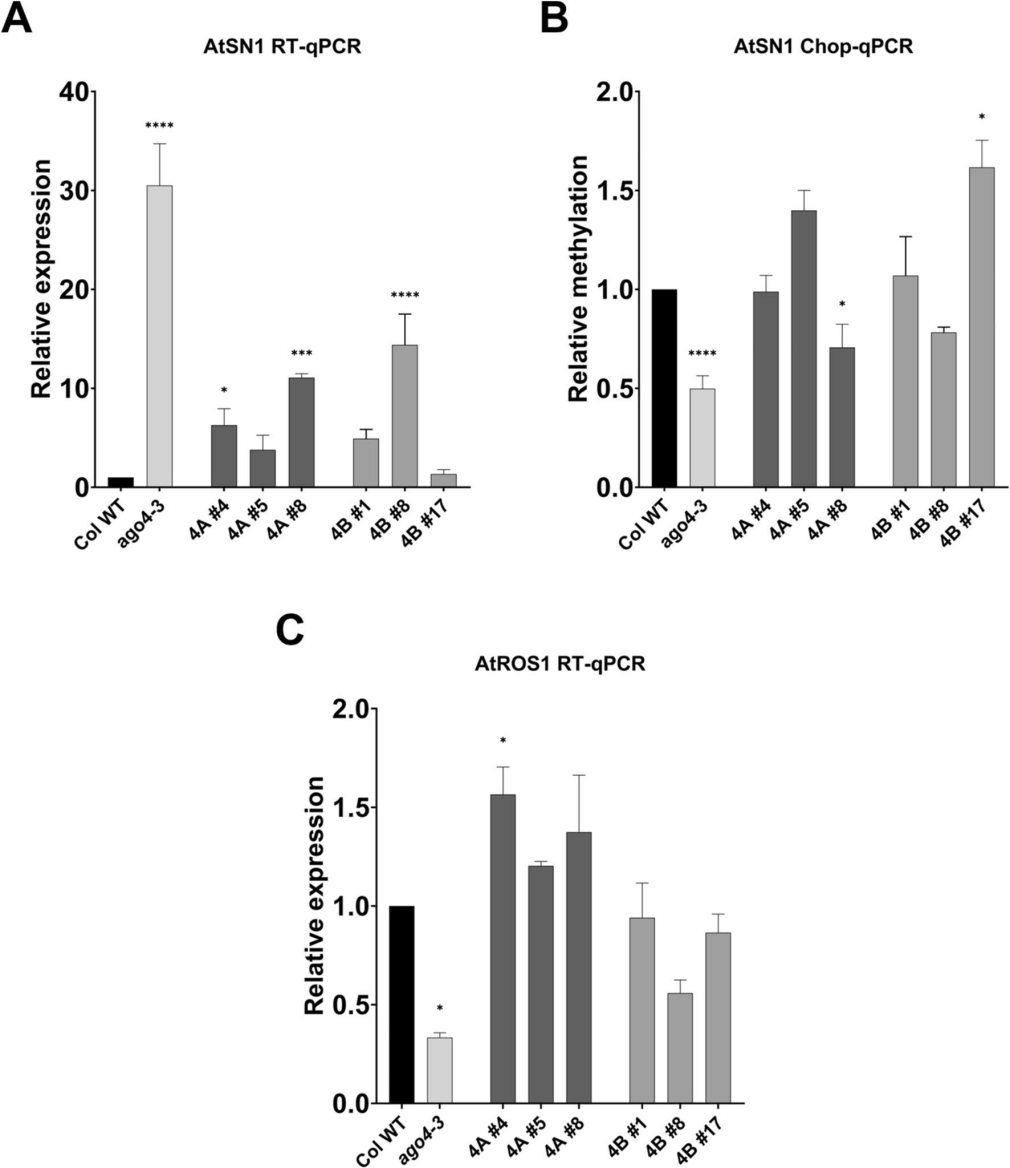
**A** *AtSN1* expression levels determined by RT-qPCR analysis of T2 mixed-stage inflorescences. Data were normalized using *AtUBC9* and *AtACT2*. **B** Methylation levels of *AtSN1* locus determined using Chop-qPCR on digested and undigested DNA. Digestion was performed with MspJI, modification-dependent restriction endonuclease, and data were normalized to *AtSN1* level from the undigested DNA and then the reciprocal was calculated to show the relative methylation levels. **C** *AtROS1* expression levels determined by RT-qPCR analysis of T2 mixed-stage inflorescences. Data were normalized using *AtUBC9* and *AtACT2*. All the results show the average of 3 independent biological replicates and were statistically analyzed against Columbia wild type (Col WT) and significant differences are indicated with asterisks (Anova one-way with Dunnett’s post-hoc test, * < 0.05, ** < 0.01, *** < 0.001 and **** < 0.0001). Error bars represent the mean ± SD, *n* = 3

The functional complementation of the barley *AGO4* genes was investigated using a retrotransposon, *AtSN1*. In *Arabidopsis*, AGO4 plays a regulatory role in the expression of *AtSN1*, influencing its transcriptional status by maintaining methylation of the locus (Zilberman et al. 2003; Havecker et al. 2010; Duan et al. 2015). In accordance with previous studies, a significant upregulation (30-fold change) of *AtSN1* expression was observed in *ago4-3*, which was successfully restricted to lower levels by HvAGO4A and HvAGO4B. The complementation effect correlated with the transgenic AGO4 content in all lines (Fig. 3A). Plants with a higher concentration of transgenic AGO4 showed an *AtSN1* expression level reminiscent to the wild type. According to the Chop-PCR, the methylation level of *AtSN1* was reduced to less than 50% in *ago4-3* mutant. As expected, the higher dosage of both barley AGO4 proteins successfully restored the methylation of *AtSN1*. Based on this finding, the reduction of *AtSN1* expression levels in the complemented lines was considered to be due to the increased methylation state at the affected locus (Fig. 3B). To further validate the functionality of the barley *AGO4* genes, the expression of *AtROS1*, a DNA glycosylase/lyase, was examined, as the dependence of the expression of this gene on RdDM had been previously demonstrated (Lei et al. 2015; Tang et al. 2016; Córdoba-Cañero et al. 2017). A 65% downregulation of *ROS1* expression was observed in the *ago4-3* mutant compared to the wild type, and this reduction was restored in the complementation lines (Fig. 3C). Interestingly, HvAGO4A plants showed even higher levels of *AtROS1* expression compared to WT, especially line 4A #4, which showed a significant upregulation (Fig. 3C). Moreover, both barley AGO4 proteins were able to compensate for the detrimental effect of the *ago4-3* mutation.

### Sequencing of the small RNA pools associated with the two HvAGO4 proteins

**Fig. 4:**
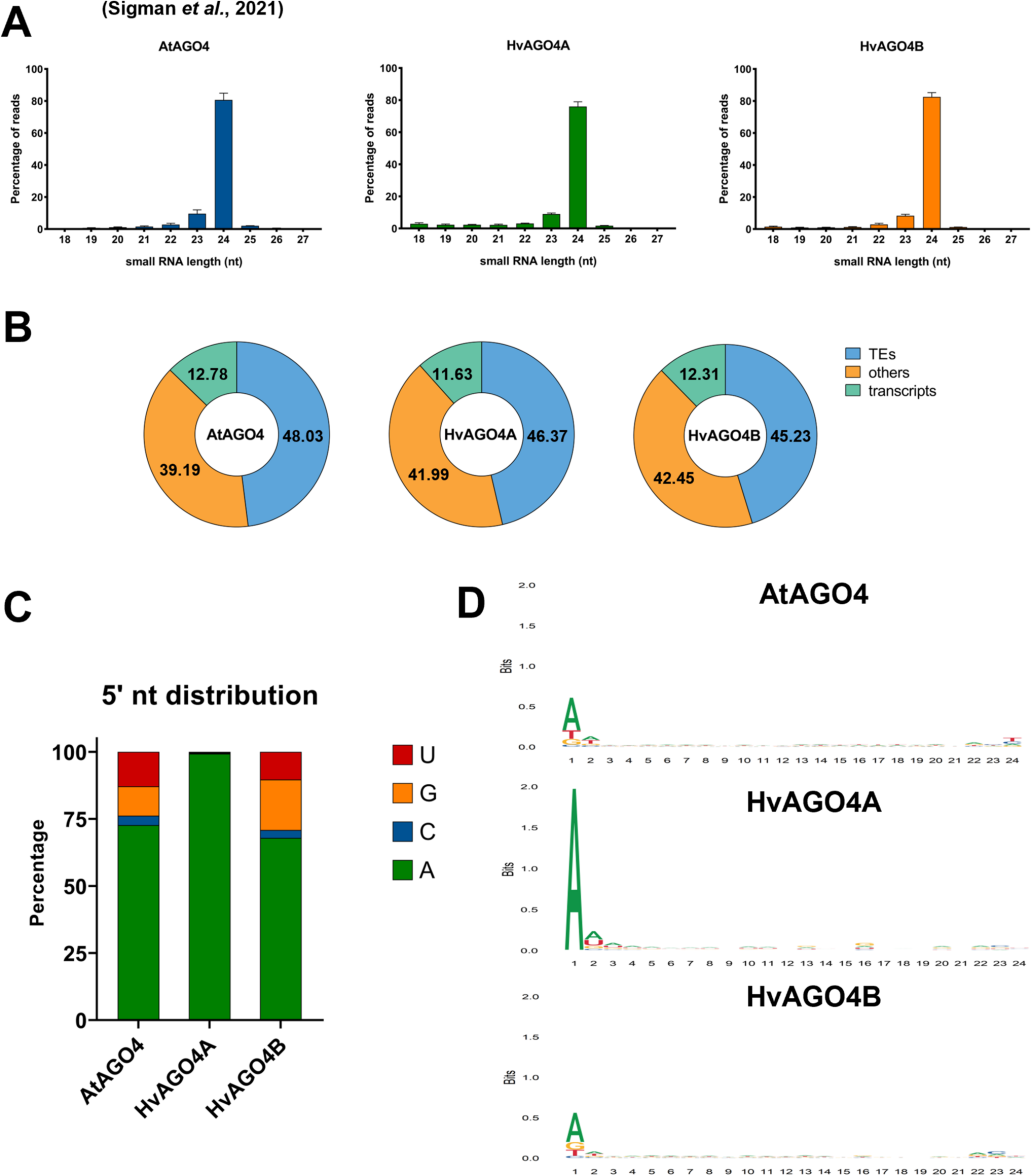
Sequencing of the HA-HvAGO4A- and HA-HvAGO4B-associated sRNA pools of *Arabidopsis* complementation plants. **A** Size distribution profile of filtered sRNA-IP data sets derived from the mean of 3 independent biological replicates. AtAGO4 IP raw data were retrieved from Sigman et al. (2021). Error bars represent the mean ± SD, *n* = 3. **B** Percentage of sRNA-IP read distribution based on their origin. **C** Percentage of the sRNA read distribution according to the 5′ nucleotide identity. **D** Graphical representation of the 24-nt long sequences conservation of nucleotides using sequence logos. The graphs represent only one data set per type; in particular, graphs of AtAGO4 #1, HvAGO4A #5, and HvAGO4B #17 are shown here. Maximum value in bits is 2 on the Y axis. Higher value for a nucleotide indicates a higher conservation

The sRNA-binding preference of AGO4 has been extensively characterized in *Arabidopsis* and rice, but no data are currently available for barley. This is particularly relevant as experiments in rice have demonstrated significant divergences in the binding preferences of sRNAs based on their 5′-end nucleotide (Wu et al. 2010). To demonstrate the sRNA binding ability of the putative HvAGO4A and HvAGO4B proteins and to determine sRNA binding similarities and differences, sRNA-IP sequencing was performed on three *Arabidopsis* complementation lines of HvAGO4A and HvAGO4B. Contamination of IP samples were checked with HA-specific western blot (Fig. S2). Raw data from an AtAGO4 sRNA-IP sequencing, obtained using a similar method to that described in the present study, were used as a control (Sigman et al. 2021). The remarkable uniformity in the size distribution pattern of the mapped sRNA reads, and the strong affinity of the putative barley AGO4 proteins for the 24-nt sRNAs suggested an orthologous function to AtAGO4 (Fig. 4A). Furthermore, comparison of the origin categories of the reads resulted in a similar distribution pattern for all the libraries. In all samples, reads originated from TEs showed the highest representation (45–48%); while, around 12% and 40% were derived from transcripts and unannotated regions of the genome, respectively (Fig. 4B). On the other hand, observing the 5′-end nucleotide distribution of sRNAs, a significant difference between HvAGO4A and HvAGO4B was observed. While both barley AGO4 proteins bind 24-nt sRNAs with adenine at the 5′-end (24A sRNAs), only HvAGO4B had a distribution pattern similar to AtAGO4, which also binds sRNAs that have either a C, G or U residue at the 5′ terminal position. In contrast to this, HvAGO4A exhibited an almost exclusive affinity for sRNAs starting with an A residue (Fig. 4C). Based on the sequence conservation analysis of the 24-nt sRNA pools retrieved from the sRNA-IP sequencing data, minor differences were detected amongst AtAGO4 and the two HvAGO4 proteins (Fig. 4D). While HvAGO4A exclusively loaded 24A sRNAs, HvAGO4B also had contact with 24-nt sRNAs having G, C, or U residues at their 5′ terminal position. Notably, the AGO4 proteins also showed a lower degree of conservation at the 3′ end. AtAGO4 displayed a loading preference for sRNAs with U residue at the 3′ terminus, HvAGO4B favored sRNAs with C residue at the 23rd nucleotide, while HvAGO4A showed no conserved position (Figs. 4D, S4A–C).

**Fig. 5:**
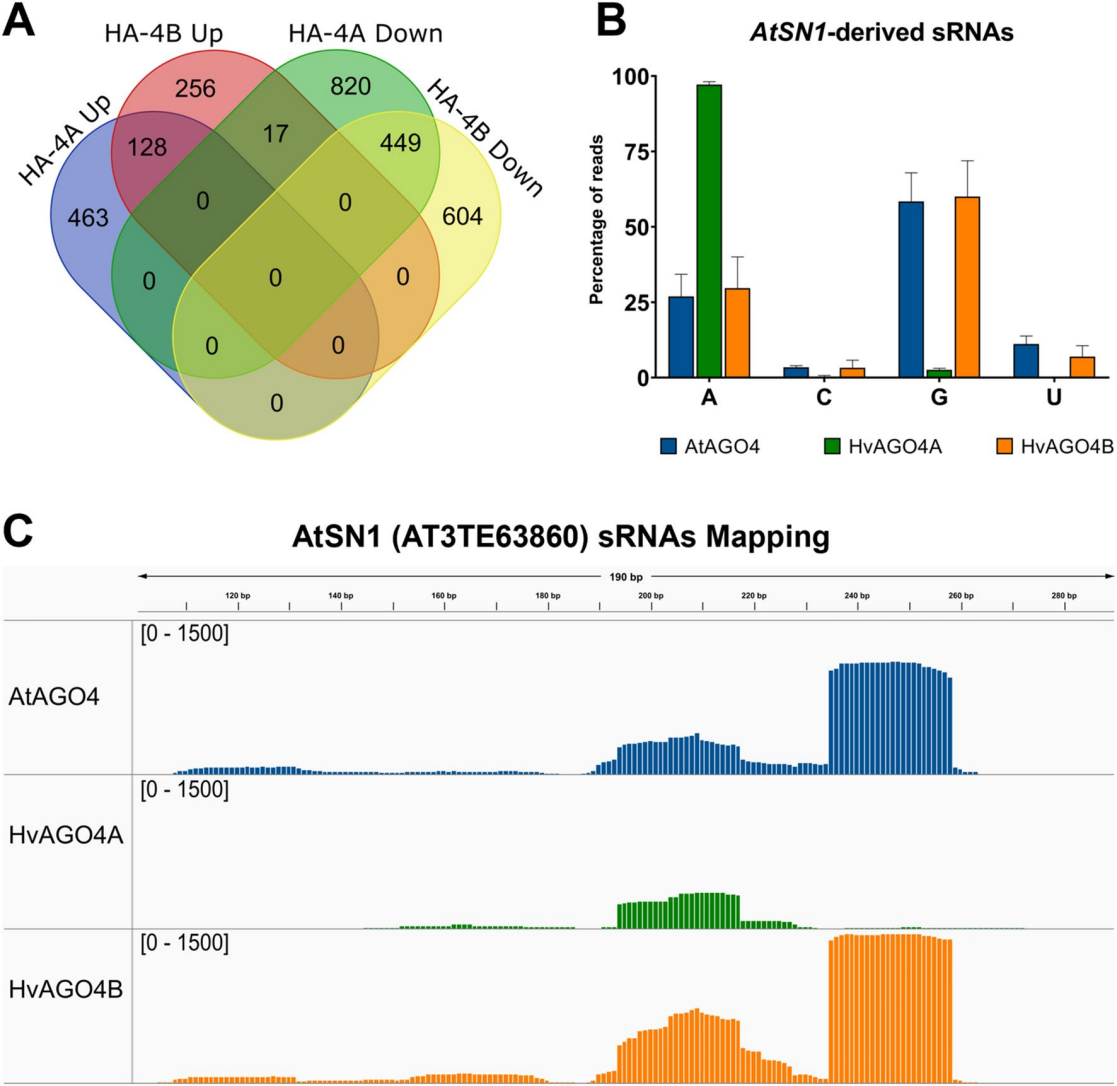
**A** Venn diagram showing the number of TEs where the amount of sRNAs show at least twofold statistically significant (*p*-value < 0.05) change compared to AtAGO4. **B** Percentage of the *AtSN1*-derived sRNAs in the categories based on 5′-end nucleotide. Error bars represent the SD of the three sequenced lines. **C** SRNAs mapped on *AtSN1* genomic locus. The reads are merged from the three independent biological replicate IP datasets. The quantity of mapped reads (counts) is written on the left side of the image inside the square brackets. Different colors indicate individual IP datasets: AtAGO4 (blue), HA-HvAGO4A (green) and HA-HvAGO4B (orange) (color figure online)

TE-derived sRNAs from HvAGO4A and HvAGO4B were compared with those from AtAGO4 to identify TEs with varying abundances of mapped sRNAs. Interestingly, 1877 TEs associated with HvAGO4A were identified, which revealed a notable discrepancy in the number of TE-derived sRNA reads compared to AtAGO4 (Fig. 5A). Of these, 591 showed higher read counts for HvAGO4A, while 1286 showed fewer reads compared to AtAGO4. In contrast, fewer distinct TEs were found in the case of HvAGO4B (1454 TEs) where mapped sRNAs exhibited either a higher (401 TEs) or a lower (1053 TEs) abundance in the IP sample of the barley protein compared to AtAGO4. Furthermore, when considering all TEs that exhibited changes in sRNA abundance in barley proteins compared to AtAGO4, 128 and 449 TEs with increased and decreased sRNA content were identified in both HvAGO4 proteins, respectively (Fig. S5A). Interestingly, 17 TEs displayed an inverse behavior, showing a higher representation in HvAGO4B and a lower sRNA abundance in HvAGO4A compared to AtAGO4. However, no TEs demonstrated the opposite trend (Figs. 5A, S5A).

In accordance with the distribution pattern of the whole pools (Fig. 4C), AtSN1-derived sRNAs also showed differences in terms of the 5′-end nucleotide preference of the different proteins. While HvAGO4A binds almost exclusively *AtSN1*-derived sRNAs beginning with A, AtAGO4 and HvAGO4B showed a higher affinity for 5′ G (around 60% for both), but with the ability to bind sRNAs with any nucleotide at their 5′ terminal base (Fig. 5B). Notably, this difference was accompanied by an altered distribution of reads mapped to the retrotransposon. AtAGO4 and HvAGO4B mainly bind to sRNAs from all four regions of the locus, while HvAGO4A had a preference for those sRNAs that were originated solely from the central region, which reduced the total amount of *AtSN1*-derived sRNAs (Fig. 5C). Analyzing the sRNAs of the different regions, a distinct distribution of 5′-end nucleotides was observed, with the central regions primarily producing sRNAs beginning with an A residue, while the two lateral regions predominantly produced sRNAs with a G residue as the 5′-end nucleotide. A closer examination of other TEs also revealed changes in the distribution pattern and end nucleotide of sRNAs. For example, when analyzing sRNAs derived from a RathE3 TE (AT5TE27090), the presence of three regions producing sRNAs in AtAGO4 and HvAGO4B was observed, but no significant amount of sRNAs was identified in HvAGO4A. Upon detailed analysis of the type of sRNAs derived from this TE, it was observed that these regions exclusively produced only sRNAs with a G residue at the 5′-end and, thus, could not be loaded by HvAGO4A (Fig. S5B). The investigation of *AtSN1* and *RathE3*, revealed that despite the similar regulatory role of the three investigated AGO4 proteins (Fig. 3A, B), there are differences in their modes of action.

### Investigation of the barley AGO4 functionality under heat stress

**Fig. 6:**
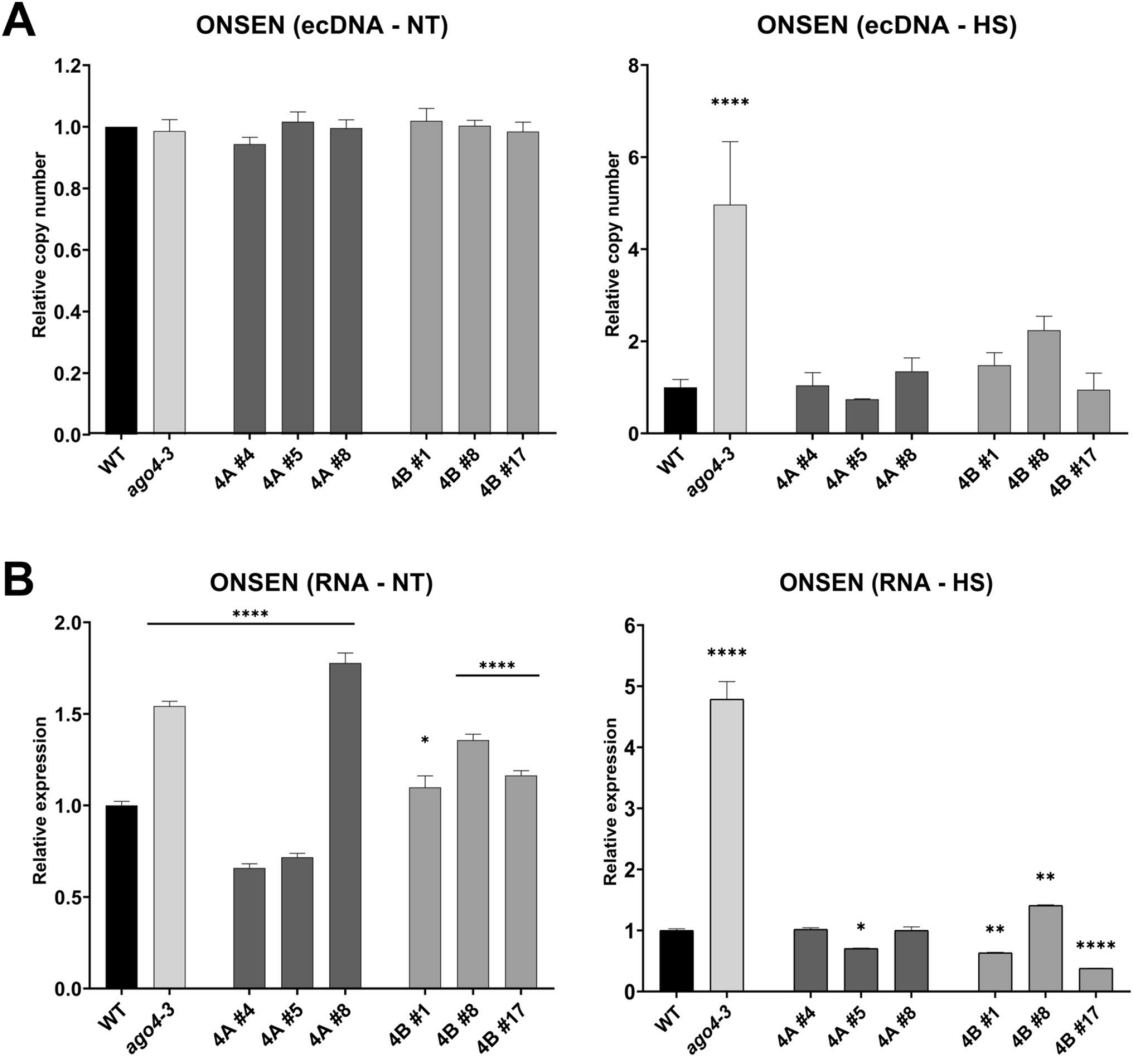
**A** Relative copy number of *ONSEN* extrachromosomal DNA (ecDNA) in non-treated (NT) and heat stress (HS—24 h at 37 °C) 1-week-old *Arabidopsis* seedlings. Data were normalized using *AtUBC9*. **B** Relative expression of *ONSEN* before and after the heat stress activation measured by RT-qPCR and normalized on *AtUBC9* and *AtPP2AA3*. Statistically significant differences compared to Col WT are indicated by asterisks (Anova one-way with Dunnett’s post-hoc test, * < 0.05, ** < 0.01, *** < 0.001 and **** < 0.0001). Error bars represent the mean ± SD, *n* = 3

TEs and genes located in their proximity may undergo transcriptional activation in response to stress conditions (Makarevitch et al. 2015; Ito 2022). To observe the effect of barley AGO4 proteins on TE activation under heat stress, 1-week-old *Arabidopsis* seedlings were subjected to 24 h heat stress at 37 °C, and extrachromosomal copy formation and transcript levels of *ONSEN*, a heat-activated retrotransposon, were examined. The extrachromosomal copy number of the 8 *ONSEN* genes in *Arabidopsis* plants grown under control conditions remained unchanged in both mutant and transformant lines compared to the wild type (Fig. 6A). In agreement with previous studies, heat stress triggered increased transposon activity in all samples (Fig. S6A), especially in the loss-of-function *ago4-3* mutant, where a significant fivefold increase in the extrachromosomal DNA (ecDNA) copy number was observed compared to wild-type samples. Both HvAGO4A and HvAGO4B reduced the activation of TEs during the stress condition when compared to the *ago4-3* mutant, maintaining the *ONSEN* ecDNA content at a similar level as to that observed in the wild type, even in the case of the lower expression transformant lines. To further demonstrate whether this phenomenon was directly related to *ONSEN* transcript expressional rate, it was investigated in non-treated (NT) and heat stress (HS) seedlings. According to the RT-qPCR analysis, *ONSEN* was significantly upregulated under heat stress conditions in the *ago4-3* mutant compared to the wild type, while all transformant lines were more similar to the wild type in terms of their *ONSEN* expression levels (Figs. 6B, S6B). Interestingly, in lines with higher transgenic protein content such as 4A #5, 4B #1, and 4B #17, *ONSEN* was even significantly downregulated compared to wild type. Under control conditions, there is a significant difference in *ONSEN* expression between transgenic, *ago4-3* mutant and wild-type plants. This change is imperceptible when compared to heat-stressed plants (Fig. S6A). In addition, under control conditions, HvAGO4A #4 and #5 had a stronger effect on *ONSEN* silencing compared to HvAGO4B.

## Discussion

The function of AGO4 in *Arabidopsis* has been extensively characterized; however, its role in monocots, especially in large genomes composed mainly of TEs, remains elusive, as do the underlying causes of its gene duplication (Duan et al. 2015; Wang and Axtell 2017; Trujillo et al. 2018). In our study, we aimed to elucidate the function of barley AGO4 proteins using *Arabidopsis* as our experimental model. We performed the analysis using heterologous complementation in *Arabidopsis* to examine both the similarities and differences between proteins belonging to the same clade. Our data suggested that HvAGO4A and HvAGO4B have similar functions to AtAGO4, primarily binding to 24-nt sRNAs (Fig. 4A) and triggering methylation at specific target loci (Fig. 3).

To identify orthologous genes or protein sequences of the RdDM pathway in barley, the model plants *Arabidopsis* and rice are commonly used, as they are among the few plants in which this mechanism has been extensively investigated. In addition, as monocotyledons, rice and barley share certain similarities in various processes. We found 4 genes belonging to the AGO4-AGO6 clade in barley: *HvAGO4a*, *HvAGO4b*, *HvAGO15,* and *HvAGO6*. These genes were previously named using in silico techniques; however, there are errors in their nomenclature, while their analysis remains incomplete (Madsen et al. 2009; Hamar et al. 2020; Yao et al. 2021). In the present study, we attempted to standardize the nomenclature and perform an in silico analysis that was as complete as possible. We primarily focused on *HvAGO4a* and *HvAGO4b*, since the expression level of *HvAGO15* was undetectable, and *HvAGO6* had functionalities that may differ from those of *AGO4* (Duan et al. 2015; McCue et al. 2015). The two *AGO4* genes in barley, orthologous to rice, showed slightly different expression levels in inflorescence tissues (Fig. 1D). Similar alterations to their expression was found in several databases, such as BaRTv1.0 and ePlant, which also confirmed, as previously reported for *Arabidopsis* AGO4, that the expression of these two genes is higher in inflorescences than in other plant tissues (Zilberman et al. 2003; Chan et al. 2004; Mascher et al. 2017; Thiel et al. 2021). Moreover, *HvAGO15*, which is characterized by minimal expression levels, contains elements with the potential to modulate its expression, such as tandem repeats in frame with the gene at the 5′-end (Wu et al. 2010; Trujillo et al. 2018). Based on the similarities observed with *AGO15* in rice, the characteristics of *HvAGO15* suggest its potential classification as a pseudogene. This observation corresponds to a recurring pattern in the genes of the AGO4 clade. Notably, AtAGO8 is classified as a pseudogene due to its significantly low expression levels, despite its membership in the AGO4 clade (Havecker et al. 2010).

The use of *Arabidopsis* for heterologous complementation of barley *AGO4* genes allowed us to investigate several loci already linked to AGO4 function. This approach enabled us to examine whether HvAGO4A and HvAGO4B could function in a similar manner to that already established for AtAGO4 at specific loci. Specifically, we selected *AtSN1* and *AtROS1* because of their previously documented transcriptional response to mutations in the RdDM pathway. Notably, *ROS1* is a gene that is finely regulated through methylation of its promoter by AGO4 (Havecker et al. 2010; Lei et al. 2015; Tang et al. 2016; Córdoba-Cañero et al. 2017). This regulation is critical for maintaining a balanced methylation level, given the antagonistic role of ROS1 toward AGO4 target loci, which often overlaps with ROS1 targets (Tang et al. 2016; Córdoba-Cañero et al. 2017). Again, our results confirmed that the expression of these two loci is indeed affected by the loss of AGO4 function in *Arabidopsis*. However, it is remarkable that barley AGO4 proteins effectively restore the expression of both loci to wild-type levels; in particular, restoration of methylation levels at the *AtSN1* locus was also observed, suggesting that these effects are highly regulated and interrelated. Our results demonstrate the high conservation of the RdDM pathway across plant species, as evidenced by the ability of barley AGO4 proteins to effectively restore pathway functionality and highlight the critical role of AGO4 in regulating DNA methylation and gene expression at specific loci, such as AtSN1 and AtROS1. This underscores the intricate regulation within the RdDM pathway, where AGO4 and ROS1, despite being in the same pathway, exhibit an antagonistic relationship, with AGO4 being able to regulate the expression of ROS1. Furthermore, our results highlight the essential and evolutionarily conserved nature of AGO4 in the RdDM pathway, which is supported by previous studies showing that AGO4 is a key regulatory protein involved in RdDM in plants.

Previous studies have demonstrated the effect of heat stress on transposon activity, implicating the involvement of the RdDM pathway (Lang-Mladek et al. 2010; Cavrak et al. 2014). While not all components of this pathway are essential for basal heat stress tolerance, loss of AGO4 in *Arabidopsis* has been observed to increase plant susceptibility to this specific form of stress (Popova et al. 2013). The *Ty1/copia*-type retrotransposon *ONSEN* (*ATCOPIA78*), known for its activation under heat stress conditions in *Arabidopsis*, showed increased transcript levels and the presence of ecDNA in mutants implicated in the RdDM pathway (Ito et al. 2011, 2013; Hayashi et al. 2020; Ito 2022). Specifically, the activation mechanism involves the recognition of a sequence within the long terminal repeat (LTR) by the heat-responsive transcription factor HsfA2 (Cavrak et al. 2014). Remarkably, under standard conditions, *ONSEN* maintains its inactivity even in mutants linked to the RdDM pathway, due to the absence of CG and CHG sites in the *ONSEN* promoter; furthermore, the reduction of DNA methylation at the CHH sites proves insufficient to activate the element (Ito et al. 2011; Cavrak et al. 2014). When evaluating the upregulation of *ONSEN* transcripts after heat stress in all plants, we observed that the basal level of *ONSEN* was extremely low under controlled conditions, showing a 2000-fold increase in the wild type and a remarkable 7000-fold increase in the *ago4-3* mutant (Fig. S6B). Our results indicated that both barley AGO4 proteins effectively restored the ecDNA and transcript levels of *ONSEN* to wild-type levels, although the *ago4-3* mutant shows elevated levels in both cases (Fig. 6A, B). In addition, the extent of *ONSEN* downregulation was found to be directly proportional to the expression levels of the introduced transgenes. This observation suggests that the presence of barley AGO4 alone is sufficient to restore the repression of TEs that become activated during periods of heat stress.

Thus far, we have highlighted the similarities among the AGO4 proteins; however, a notable divergence emerged in their affinity for the 5′-end nucleotide of sRNAs. HvAGO4B shares its main characteristics with AtAGO4 and OsAGO4B, both of which have a predisposition to bind sRNAs with a 5′ A residue but retain the ability to bind sRNAs with 5′ G or U residues (Fig. 4C). In contrast, HvAGO4A appears to exclusively bind sRNAs with a 5′ A residue. A similar distinction is clear in rice when comparing OsAGO4A and OsAGO4B (Wu et al. 2010). Additionally, similar differences can be noted with *Arabidopsis* AtAGO6 and AtAGO9, which unlike AtAGO4, both exhibit a strong loading preference for sRNAs with a 5′ terminal A residue with percentages of 94% and 97% in AtAGO6 and AtAGO9, respectively (Havecker et al. 2010). Previously, studies have been undertaken concerning the structure of the MID domain of Argonaute proteins, highlighting its responsibility for the selective recognition of the 5′ nucleotide (Frank et al. 2012). Interestingly, a coordination between the MID and PIWI domains has been identified as the underlying reason for this specificity (Liu et al. 2022). In our study, we found that, among the conserved sites involved in 5′ nucleotide anchoring, only one site differed between AtAGO4, HvAGO4A and HvAGO4B, which was conserved between rice and barley (Fig. 1C). This site consists of four amino acids (QCxA), and the third amino acid could potentially be a determining factor of the specificity at the 5′-end of sRNAs. In the case of AGO6, this site does not change between *Arabidopsis*, rice or barley, maintaining a QCIx sequence (Fig. S3A); in fact, no differences in specificity are observed between *Arabidopsis* and rice (Havecker et al. 2010; Wu et al. 2010). Taking this into account, in monocots, the duplication of the *AGO4* gene could lead to the specialization of AGO4A resulting in hybrid characteristics between AtAGO4 and AtAGO6, as it resembles the former in length selection and the latter in affinity for sRNAs, beginning with an A residue. Indeed, in *Arabidopsis*, AGO6 was found to be able to load 24-nt sRNAs, as well as shorter sRNAs (19–22 nt), but with an exclusive preference for sRNAs with A residues at their 5′ ends. These length- and nucleotide-specific features ensure non-competition with other AGO proteins, particularly AGO1, which primarily binds miRNAs characterized by a length of 21 nucleotides and a starting base of U (McCue et al. 2015; Liu et al. 2022). This diversification might play a more specialized role in barley and tissues where both AGO4 proteins are expressed, potentially involving distinct localizations at the cell type level between the two.

In our study, we delineated the distinct binding capacities of HvAGO4 and HvAGO4B proteins in barley regarding sRNAs in a general context. While both barley AGO4 proteins bind specific TE-derived sRNAs, we observed exclusive binding by HvAGO4A in specific cases, which was predominantly determined by the first nucleotide of the sRNA sequence. Notably, our analysis revealed differential active sRNA production patterns within specific regions of the *AtSN1*. The central region predominantly produces sRNAs starting with an A residue, whereas the two lateral regions, particularly the 3′-end terminal segment of the locus, produce sRNAs with G or U residues at the 5′-end (Fig. 5A, B). In this specific context, the different profiles of AGO4-bound, TE-derived sRNAs induce a remarkable difference in the cumulative sRNA abundance mapped to *AtSN1*, especially in the case of HvAGO4A when compared to the other two proteins (Fig. 5C). A similar trend is observed for another TE, *AT5TE27090*, which belongs to the RathE3 family (Fig. S5B). Notably, the number of TE-derived sRNAs bound to HvAGO4A was almost negligible. This observation highlights the possibility of HvAGO4 proteins having at least partially distinct regulatory properties on TEs, especially on relatively short TEs which cannot produce sRNAs suitable for all AGO4-clade proteins. In contrast, the versatile binding properties of AtAGO4 and HvAGO4B may provide a functional advantage over those AGO proteins that bind sRNAs with absolute specificity regarding to sRNA 5′ terminal nucleotide.

In conclusion, our study of the function and behavior of barley AGO4 proteins in *Arabidopsis* heterologous complementation has provided insights into their distinct binding abilities, specificities, and involvement in TE regulation. The observed duplication of *AGO4* genes, facilitating specific behaviors while preserving core functionality, underscores an evolutionary advantage within the plant. Furthermore, a comprehensive understanding of their precise functions and potential cell type-specific localization requires further investigation in barley.

### Supplementary Information

Below is the link to the electronic supplementary material.

Supplementary file1 (PDF 737 KB)

Supplementary file2 (XLSX 14 KB)

## Data Availability

Raw data from the RNA sequencing of barley cv. Golden Promise 15–25 mm spikes have been deposited in the NCBI BioProject database under the ID PRJNA1052601 with the SRA accessions SRR27206020-SRR27206022. Raw data from small RNA-IP sequencing of HA-HvAGO4A and HA-HvAGO4B have been deposited in the NCBI BioProject database under the ID PRJNA1052470 with SRA accessions SRR27205628- SRR27205633.
