# Supplementary material for: Barley AGO4 proteins show overlapping functionality with distinct small RNA-binding properties in heterologous complementation
Source: Plant Cell Rep. 2024 Mar 13;43(4):96. doi: 10.1007/s00299-024-03177-z (PMC10937801; doi:10.1007/s00299-024-03177-z)
Supplement: Supplementary file 1 — Supplementary file1 (PDF 737 KB) [file 299_2024_3177_MOESM1_ESM.pdf]

Barley AGO4 proteins show overlapping functionality with distinct small RNA binding properties in heterologous complementation

Fabio Miloro, András Kis, Zoltán Havelda and Ágnes Dalmadi

Supplementary data

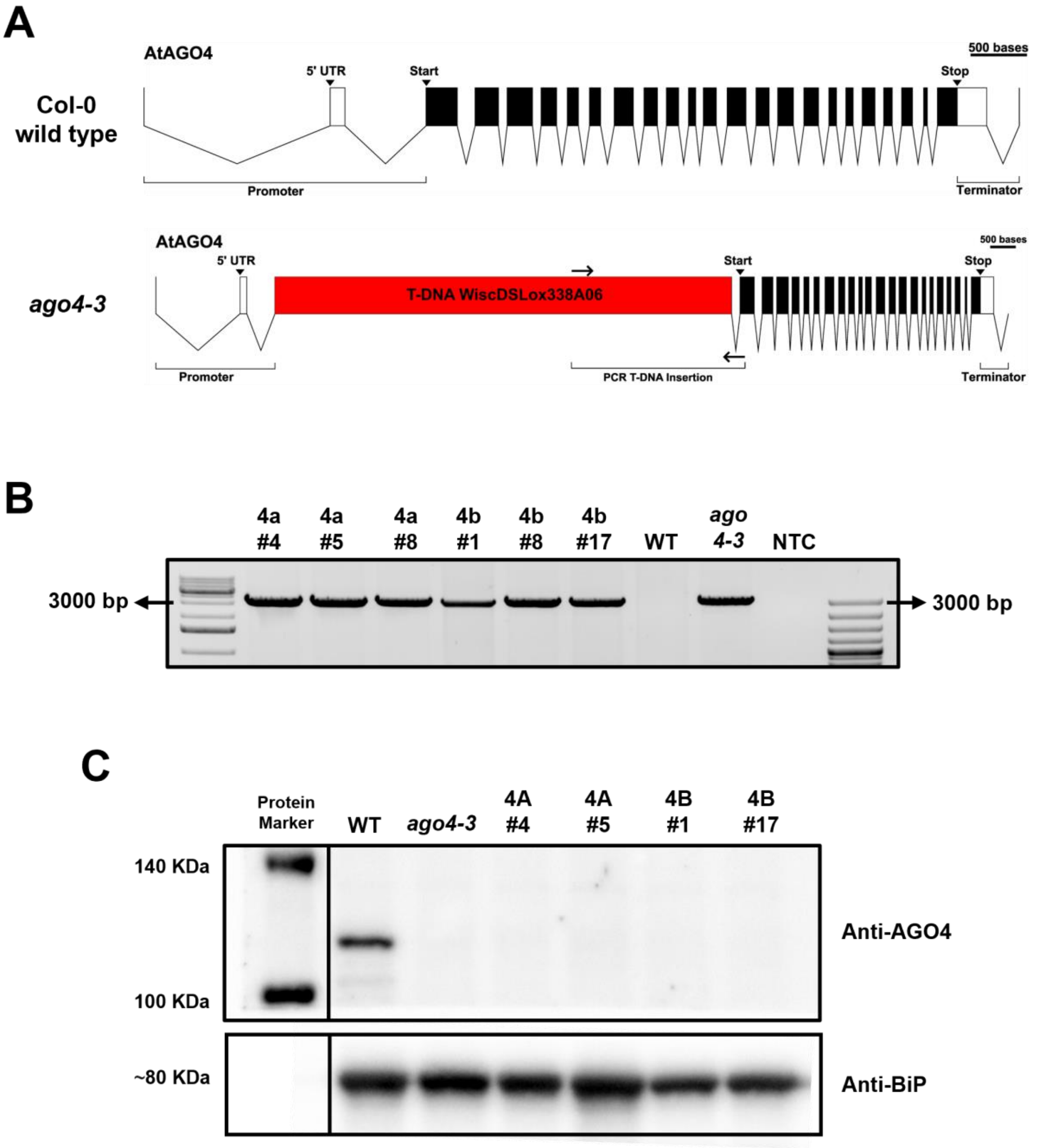

Figure S1 A. Visualization of the *AtAGO4* gene structure in wild type (top) and in *ago4-3* mutant (bottom). The promoter, 5' UTR, start and stop codons, and terminator are shown. T-DNA insertion between the 5' UTR and the start codon in *ago4-3* is marked with red. The UTR regions are demonstrated with empty shapes. The primers for the amplification of the region containing the T-

DNA are represented by arrows. The scale bars represent 500 bp. **B.** Agarose gel electrophoresis of PCR products from the region containing the T-DNA insertion in the *ago4-3* mutant. *ago4-3* and all lines derived from the *ago4-3* background are positive for T-DNA insertion, while wild-type plants are negative. The forward primer is located on the T-DNA, specifically on the phosphinothricin resistance gene, while the reverse primer is located on the first coding exon, just after the start codon. The PCR product is 3405 bp and contains a fragment of the phosphinothricin resistance gene, its promoter, Ds transposon, LoxP site, T-DNA Left Border and a 170 bp promoter fragment before the start codon. The first lane is loaded with GeneRuler 1 kb Plus DNA Ladder and the last lane is loaded with GeneRuler 100 bp Plus DNA Ladder. NTC is for No Template Control. **C.** Western blot showing the endogenous protein level of AtAGO4 in wild type, *ago4-3* mutant and the lines with the highest level of barley HA-tagged AGO4 protein. The first lane is loaded with ProSieve QuadColor Protein Marker (Lonza Bioscience), which gives an aspecific signal to the anti-AtAGO4 antibody. The membrane was cut into two, and lower part was used to detect BiP (lumenal-binding protein) as an internal control. The same membrane was divided in two and incubated each half separately with the two different antibodies.

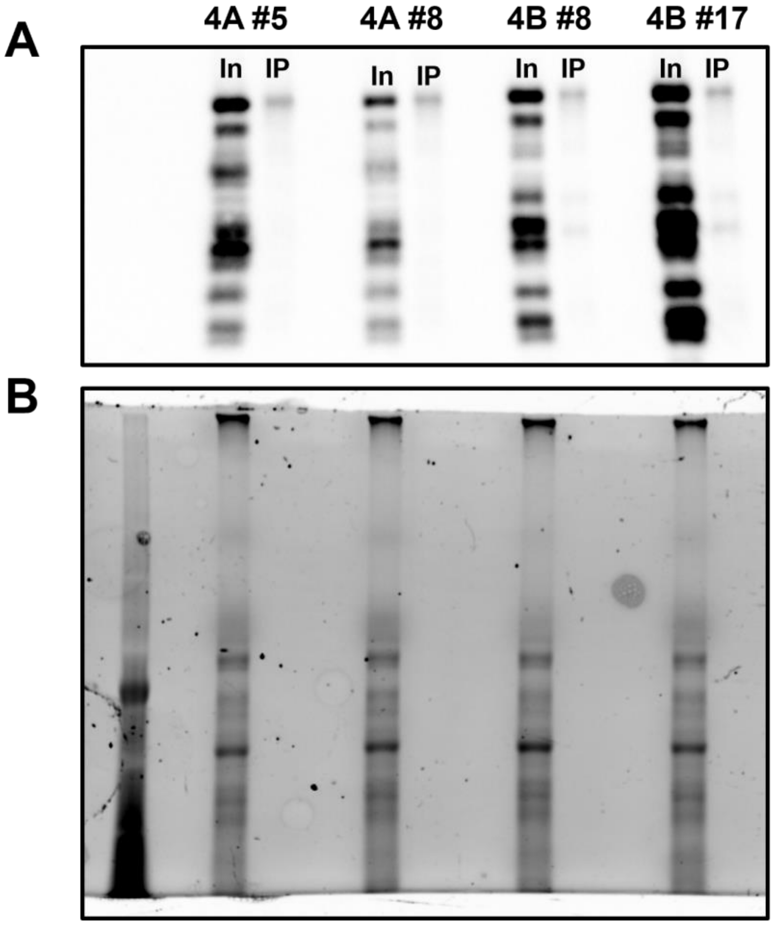

Figure S2 A. Western blot showing the protein expression of the barley HA-tagged proteins (HA-HvAGO4A and HA-HvAGO4B) used for small RNA-IP sequencing. The first lane is loaded with ProSieve^{TM} QuadColor^{TM} Protein Marker (Lonza Bioscience). The other lanes are loaded so that the left lane contains the input (In) from the protein extraction before immunoprecipitation and the right lane contains the sample after immunoprecipitation with anti-HA (IP). The membrane was exposed for 5 seconds. **B.** The gel on which the samples were run was a Mini-PROTEAN TGX Precast Protein Gel (BioRad) and shows the total amount of proteins present in the different lanes after UV cross-linking.

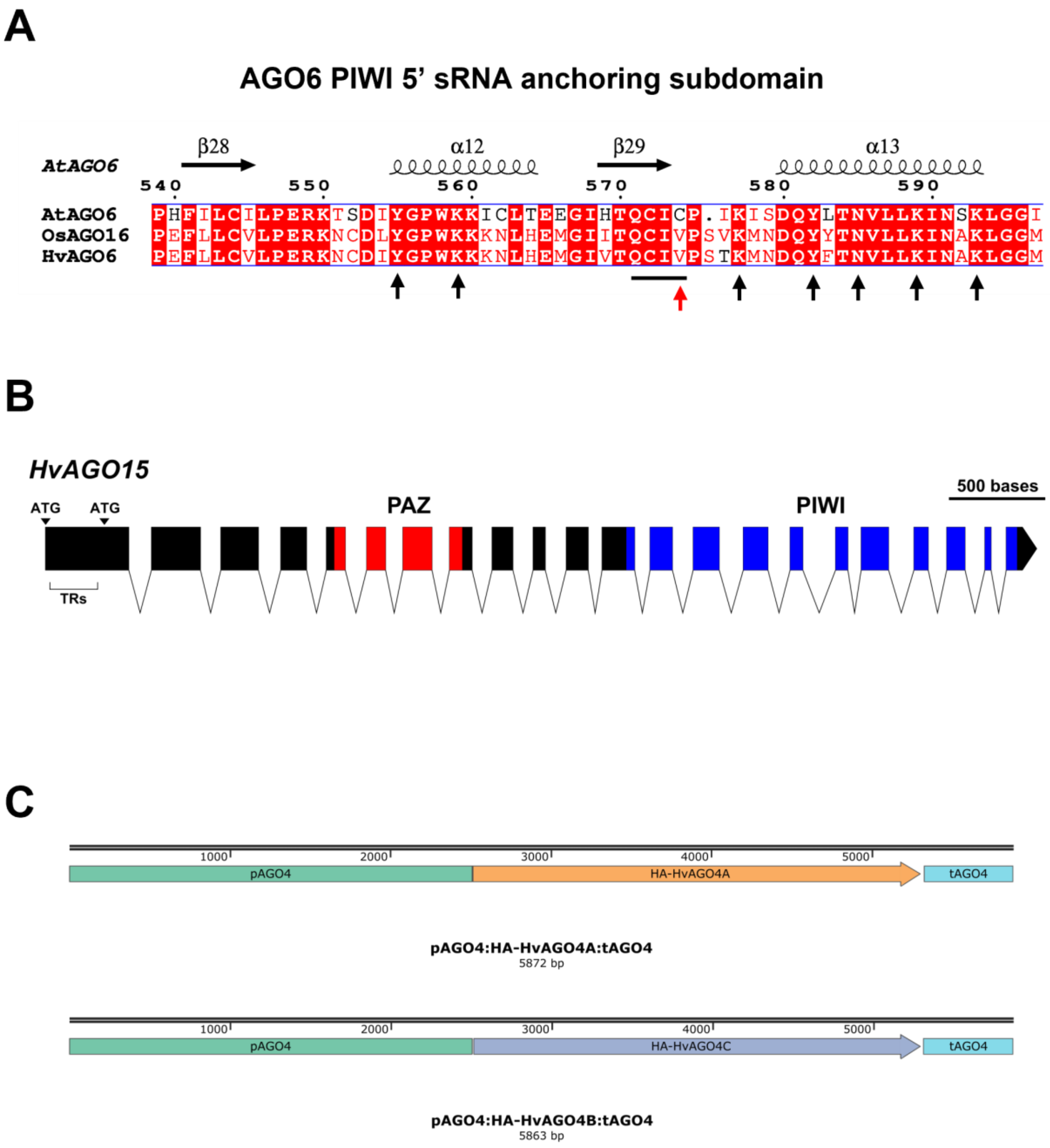

Figure S3 A. Protein alignment of the PIWI domain region involved in the 5' sRNA anchoring of the Arabidopsis, rice and barley AGO6 proteins. Alignment was performed with ClustalW and was visualized using ESPript. The vertical black arrows and the black line indicate the amino acids directly involved in the binding domain, while the red arrow indicates the AA showing variations between Arabidopsis (dicot) and the monocots. **B.** Visualization of the *HvAGO15* gene structure. Solid rectangles and lines indicate exons of the coding regions and the introns, respectively. The different possible transcriptional starts are indicated with ATGs, and the region with possible regulatory elements to control expression is marked with TRs (tandem repeats). Within the coding region PAZ and PIWI domains were labelled with red and blue, respectively. The scale bar represents 500 bp. **C.** Schematic representation of expression cassettes used to generate transgenic plants for heterologous complementation. The top line indicates the size and position of each element. Green and light blue are the promoter and terminator of the Arabidopsis AGO4 gene, respectively. The ORF in between is the HvAGO4a or HvAGO4b gene with 5' HA epitope attached.

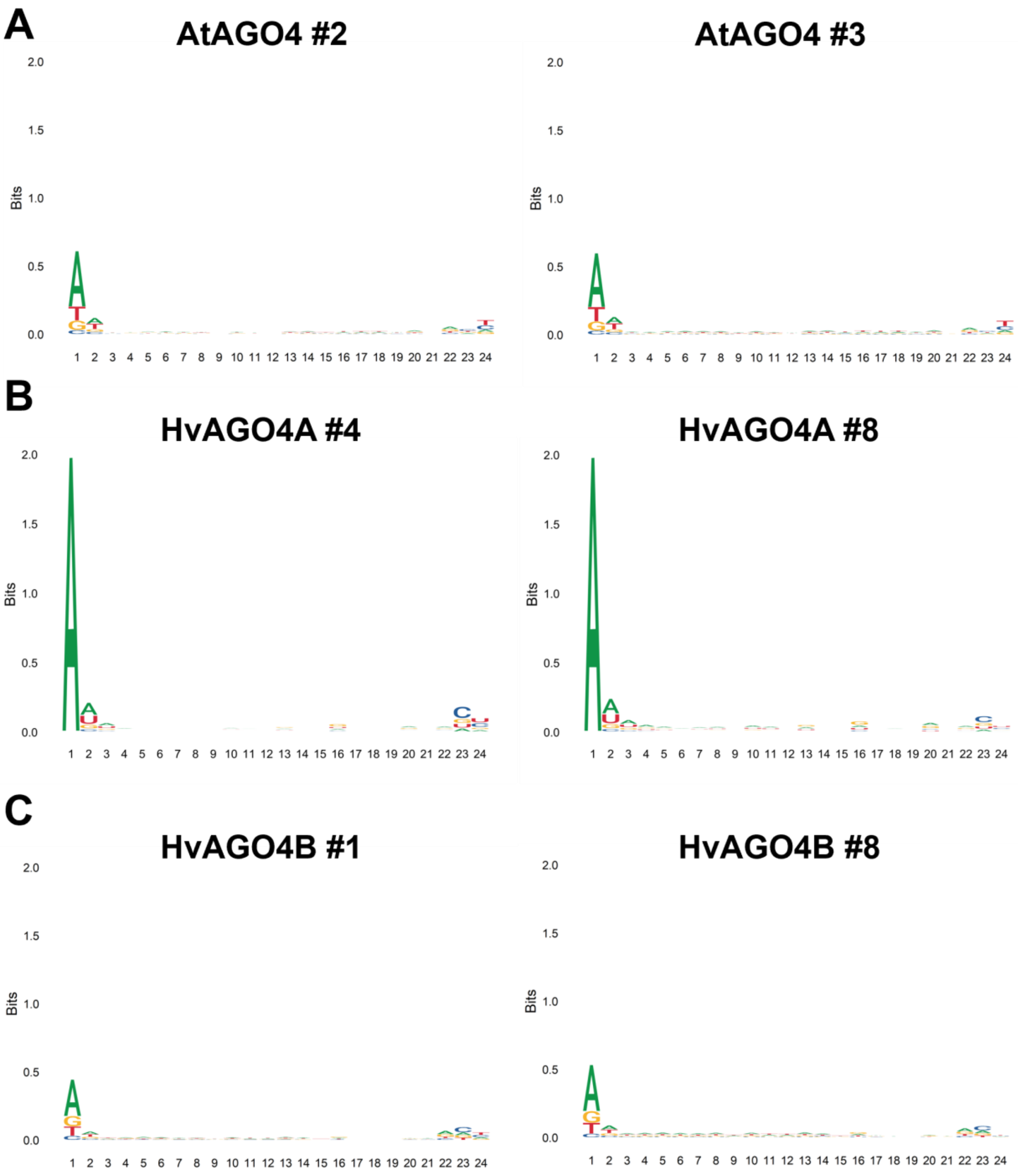

Figure S4 A-C Graphical representation of the nucleotide conservation in 24-nt long, AGO4-associated sequences using sequence logos in all the other small RNA-sequencing not shown in the main text. Maximum value in bits is 2 on the Y-axis and the range is the same for all the graphs. A higher value for a nucleotide indicates higher conservation.

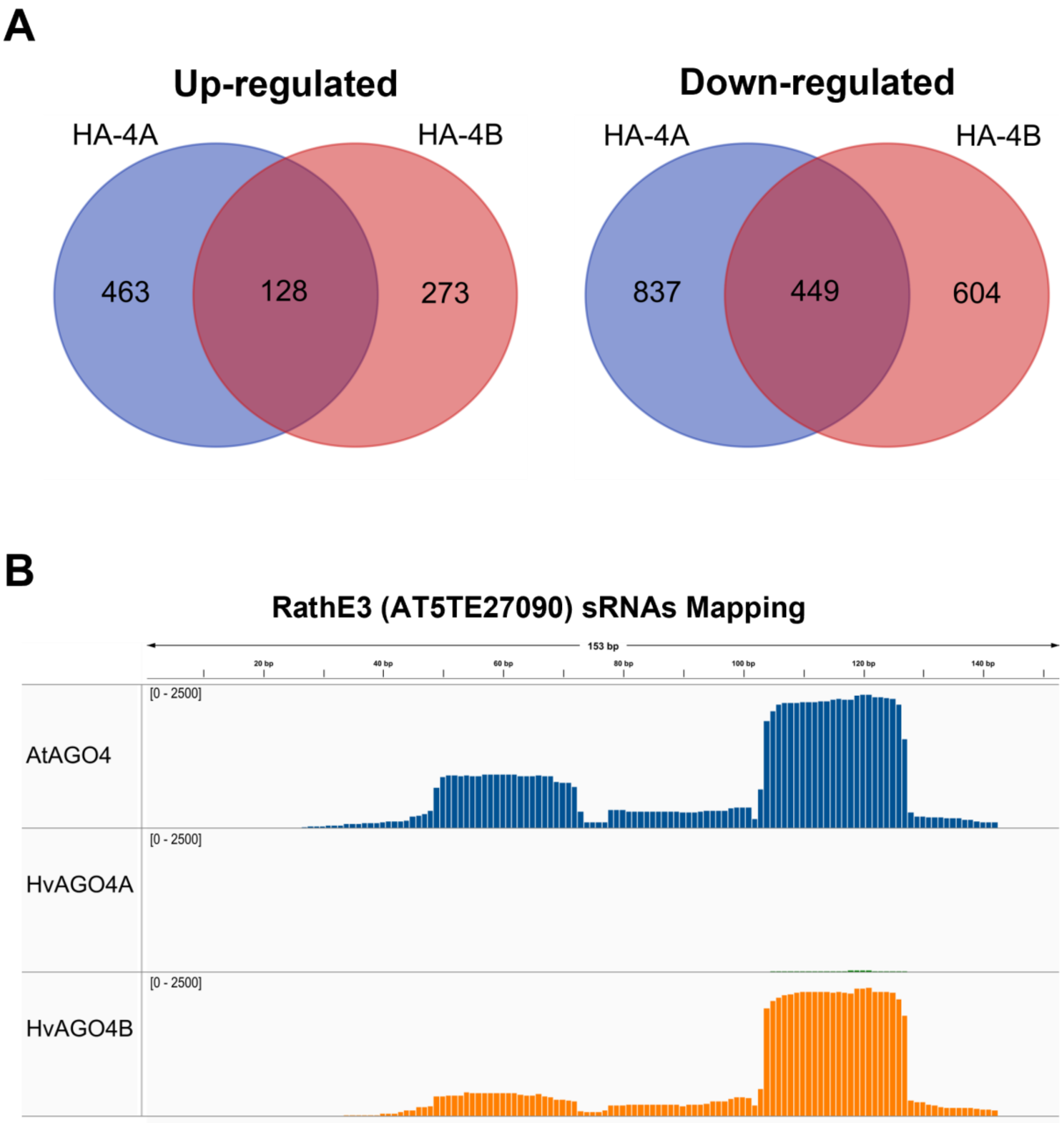

Figure S5 A. Venn diagrams illustrating the number of transposable elements where the amount of small RNAs shows at least a 2-fold statistically significant change (p-value<0.05) compared to AtAGO4, on the left the up-regulated and on the right the down-regulated. **B.** Small RNAs mapped on the genomic locus of one member of RathE3 TE family (*AT5TE27090*). The range of mapped reads (counts) is written on the left side of the image inside the square brackets, and it is the same for all the 3 datasets. Different colors indicate individual IP datasets: AtAGO4 (blue), HA-HvAGO4A (green) and HA-HvAGO4B (orange).

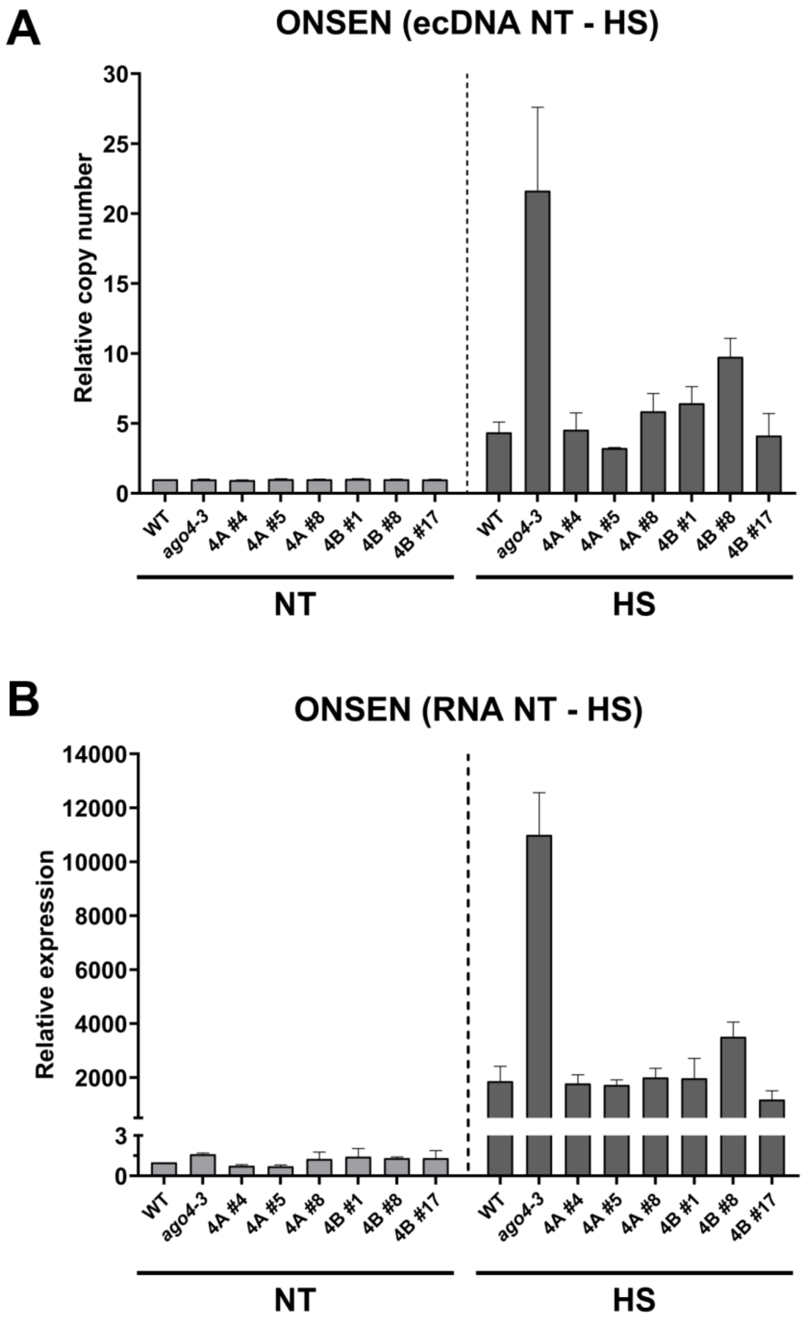

Figure S6 A. Relative copy number of *ONSEN* extrachromosomal DNA (ecDNA) in non-treated (NT) and heat stress (HS - 24 hours at 37 °C) 1-week old Arabidopsis seedlings. Data was normalized using *AtUBC9*. **B.** Relative expression of *ONSEN* before and after the heat stress activation measured by RT-qPCR and normalized on *AtUBC9* and *AtPP2AA3*. For both the graphs, WT in the NT group has been set as 1 for all the other samples, and the dotted line divides the graph between NT and HS samples. Both the graphs show the data already presented in the main text but using the same scale to show the real difference between non-treated and heat stress values.
